# Effects of Age, Fulton’s Condition Index (K) and Muscle Fat on Total Mercury Content in Raw, Pre-Canning and Canned Samples of Atlantic Bluefin Tuna (*Thunnus thynnus*)

**DOI:** 10.3390/foods12142686

**Published:** 2023-07-12

**Authors:** Pierluigi Piras, Nicolò Pietro Paolo Macciotta, Domenico Meloni, Andrea Sanna, Maurizio Cossu, Severyn Salis, Giannina Chessa

**Affiliations:** 1 DVM Practitioner, Independent Researcher, 09016 Iglesias, Italy; pirasp@tiscali.it; 2 Department of Agricultural Sciences, University of Sassari, 07100 Sassari, Italy; macciott@uniss.it; 3 Department of Veterinary Medicine, University of Sassari, 07100 Sassari, Italy; 4 Environmental Chemistry Unit, Istituto Zooprofilattico Sperimentale della Sardegna, 07100 Sassari, Italy; andrea.sanna@izs-sardegna.it (A.S.); maurizio.cossu@izs-sardegna.it (M.C.); severyn.salis@izs-sardegna.it (S.S.); giannina.chessa@izs-sardegna.it (G.C.)

**Keywords:** mercury, Atlantic bluefin tuna, fixed trap, age, Fulton’s condition index (K), fat

## Abstract

A total of 30 samples of Atlantic bluefin tuna were analysed for total mercury concentration. Relationships between total mercury content and age, Fulton’s condition index (K) and fat content were statistically evaluated. The effect of muscle status (raw, pre-canning, canned) on mercury content was also investigated. The average total mercury content was: 1.185 ± 0.662 mg/kg in raw, 1.481 ± 0.893 mg/kg in pre-canning and 1.393 ± 0.882 mg/kg in canned samples, respectively. Canning promotes a statistically significant increase in the concentration of mercury. The weight of fish, K and fat content are useful tools to estimate the mercury accumulation in Atlantic bluefin tuna. The results of the present study represent a contribution to the assessment of the EU mercury levels in Atlantic bluefin tuna.

## 1. Introduction

The Scombridae family, including tuna, show a wide range of variability in life history attributes such as longevities, growth rates, maturity, sizes, and habitat [1]. In particular, the bluefin tuna species, which are the largest and longest-lived species of scombrids, are characterized by slow growth rates, late maturation (103–159 cm and 4–9 years) and short spawning seasons. The term “bluefin tuna” indicates three species which are very similar to each other. In particular, the two northern hemisphere species [2] were recently unified as “Northern bluefin tuna” [3]. Therefore, in addition to the current “Atlantic bluefin tuna” or *Thunnus thynnus* [4], we can then consider the restored “Pacific bluefin tuna” or *Thunnus orientalis* [5], as well as the southern hemisphere congener, the “Southern bluefin tuna” or *Thunnus maccoyii* [6]. Large predators, such as bluefin tunas, are at the top of aquatic food chains, and hence they can accumulate large amounts of heavy metals. In addition, these greatly advanced pelagic fishes show high performances and very high metabolic rates. Consequently, their food intake rate is high, contributing to the accumulation of these pollutants. Among heavy metals, mercury is a ubiquitous contaminant due to its global persistence in aquatic environments. Mercury presents a relatively long biological half-life (2 years or more), can accumulate within biological tissues, and tends to bio-magnify within trophic food chains. Positive linear relations between mercury and age, or weight, have all been consistently reported among many predatory fishes, so that size can be used to estimate the mercury burden of fish and to guide decisions on their consumptive value. Previous studies [7,8,9,10,11,12] have been specifically targeted to determine the levels of total mercury in the muscle tissue of Atlantic bluefin tuna (*Thunnus thynnus*) caught or reared in the Mediterranean Sea in order to assess whether they exceed the EU official levels of 1.0 mg/kg wet weight [13]. All these studies reported that bluefin tuna samples exceeding the EU official level ranged between 26% and 100%. Several biotic factors (age, diet, metabolism) may affect heavy metal accumulation in fishes, and literature on bioaccumulation emphasizes the importance of biometric parameters above all. As a matter of fact, mercury concentration in tunas increases with body size, suggesting that mercury levels increase as these fish grow [14]. Mercury concentration is uniform in the tissues of several fish species. However, bluefin tunas (*Thunnus* spp.) are well recognized as having distinct groups of tissues [1,2,3,4,6,7,8,13,15,16,17,18,19,20,21,22,23,24], easily identifiable based on lipid content, colour, location, and muscle structure [25]. Monitoring of mercury concentration in caught and farmed bluefin tunas shows a reduction in mercury concentration in farmed fishes. The primary aim of tuna farming is to rapidly increase the biomass and lipid content of tissues, as well as to supply fish markets all year round. Moreover, bluefin tuna farming may help Food Business Operators (FBOs) to manage mercury residue levels. The high lipid content in farmed Pacific bluefin tunas affects the mercury distribution: when the lipid content is extremely high, the total mercury concentration decreases [23]. Despite differences in fish size and in Fulton’s condition index (K), the mercury concentration in tissues of Southern bluefin tunas decreases with increasing lipid content, which shows a dilution effect on mercury [15]. Therefore, the mercury concentration is negatively correlated with the lipid concentration of tissues: fatty tissues show a lower mercury concentration than lean tissues [25]. In Southern bluefin tunas, over a farming period of approximately 18–20 weeks, growing resulted in a reduction of mercury levels in edible tissues. However, a more extended farming could increase mercury concentration due to the combined effects of mercury accumulation and seasonal lipid depletion [16]. Several morphometric changes during the fattening of Atlantic bluefin tuna have been previously reported [26] with heavy weights and increase in somatic conditions of fattened bluefin tunas over 180 cm of Straight Fork length (SFL). Smaller bluefin tunas do not seem to be as influenced by the fattening process, since they are in a growth phase and their metabolic rates avoid them to become overweight. According to a previous study [7], a high difference in weight between wild and reared Atlantic bluefin tuna with the same FL has been pointed out. The age of tunas has a significant impact on K. Older fish (10–20 years old) show higher values than younger ones (5–9 years old). An evident faster growth was highlighted in 5–8-year-old Atlantic bluefin tunas. Subsequently, the growth rate decreased in terms of length and increased in terms of fattening [27]. Another study [22] examined size, K and age of Atlantic bluefin tunas in relation to contaminant levels. Farmed tunas in sea cages had lower concentrations of heavy metals in muscle tissue than wild tunas, suggesting that the fattening process may decrease the mercury concentrations. The same authors [8] studied the levels of total mercury in muscle tissue of Atlantic bluefin tuna reared in the Mediterranean Sea in relation to biological parameters and rearing period, showing a significant relationship with the size and age of tunas. In addition, it was clearly demonstrated that the longer the rearing period, the lower the mercury concentration [8]. Even more evident results were reported [28] on mercury concentration in farmed and wild Atlantic bluefin tuna caught in the Mediterranean area. Mercury content in farmed tunas was always below the EU official limits, whereas the wild ones showed a mercury content over the EU official limits. Together with the size of bluefin tunas, it seems interesting to discover if further positive linear relations between mercury, age and weight can be used by the Competent Authority (CA) to estimate their mercury burden. For example, other important relationships between mercury levels and K, which is an estimate of the fattening state of the specimens, should be taken into consideration. This could be useful as well in end-of-cycle evaluations of farmed tuna, also as a screening tool in tuna traps to decide on the immediate suitability of wild tunas for food consumption or for an appropriate cycle of fattening in aquaculture. A few studies investigated the evolution of mercury concentration in the same samples of tuna during the canning process in oil, in particular in albacore tuna or *Thunnus alalunga* [17] and in skipjack tuna or *Katsuwonus pelamis* [4]. Mercury concentration in albacore tuna samples increased significantly from raw samples to canned samples [29], but changes in mercury concentration were not directly correlated with changes in lipids on an individual basis. Other authors [30] followed the behaviour of the mercury content during the canning process in skipjack tuna muscle samples to highlight the changes in its concentration, considering three distinct phases (raw, cooked, and canned tuna). The overall results indicate an increase in the concentration of mercury during the process. Since mercury can bind to sulfhydryl groups (typically cysteine), it is possible for mercury concentrations to change as the lipid or moisture content of the muscle changes during processing. To the best of our knowledge, there are no available studies that investigated changes in the concentration of mercury in the same samples of individual specimens of Atlantic bluefin tuna during the canning process in oil. For these reasons, the aims of the present study were: (a) to measure mercury concentration in the raw tissues of Atlantic wild bluefin tunas and to assess the contamination in relation to age class, absolute and relative weight of the specimens expressed with K as well as the fat content (%) of the samples; (b) to investigate changes in the concentration of mercury in the same samples of individual specimens of Atlantic bluefin tuna during the canning process in oil; (c) to analyse the effects of canning on the mercury concentration and determine eventual statistical correlations between mercury concentration and its changes in relation to the original lipid content.

## 2. Materials and Methods

### 2.1. Sampling

Our study was carried out on samples from a batch of 271 specimens of Atlantic wild bluefin tuna caught on the fishing day of 25 May 2020 in a fixed trap of the company Tonnare Sulcitane s.r.l. located in the Municipality of Portoscuso, south-western coast of Sardinia (Italy). Each of the 271 tunas was supplied with a band with individual identification number, relating to the Bluefin-tuna Catch Document BCD-IT20900711-batch 123, as provided by the International Commission for the Conservation of Atlantic Tunas (ICCAT). In order to comply with the official methods of sampling and analysis for the control of the levels of contaminants in foodstuffs [18], a division in homogeneous sub-lots in relation to size and age of the specimens was carried out. The correlation between size and age of tuna was evaluated according to previous studies [8,9,21,22,23,24,25,29,31,32]. It was found that 78.6% of the batch (n. 213 specimens) consisted of “very young” tuna, estimated to be up to 5 years old (weight range: 20.2–55.4 kg, mean ± standard deviation (s.d.): 38.4 ± 7.9; length range: 102–146 cm, mean ± s.d. 129 ± 10). A total of 23 specimens were classified as “less young”, with an estimated age ranging between 6 and 10 years (weight range: 57.2–117.2 kg, mean ± s.d.: 72.1 ± 19.1; length range: 149–183 cm, mean ± s.d. 160 ± 10) while a further subset of 18 specimens was classified as “adult” tuna, with an estimated age of 11 to 15 years (weight range: 125.6–258.0 kg, mean ± s.d.: 200.6 ± 45.2; length range: 185–237 cm, mean ± s.d. 218 ± 19). The remaining 17 specimens were classified as “decidedly mature”, with an estimated age of between 16 and 20 years (weight range: 261.0–372.0 kg, mean ± s.d.: 307.1 ± 33.1; length range: 242–265 cm, mean ± s.d. 254 ± 9). Considering that the bioaccumulation of mercury is also a function of the exposure time, therefore of the age of the fish [33], we evaluated the correlations in three out of four age groups, i.e., tuna over 5 years of age, and selected a subset of 10 specimens for each of the estimated age groups, for a total of 30 deceased specimens (Table 1). Each specimen was identified with the individual identification number (Cod ID) applied during the fishing day. All the 30 tuna specimens were weighed individually and were measured longitudinally to obtain the “curved fork length” (*CFL*) from the upper jaw (end of the snout) to the fork of the caudal fin. Subsequently, the “straight fork length” (*SFL*) was derived through the following formula:

(1) SFL=0.9766×CFL – 2.0621 

according to previous studies [19]. Through the relation between the whole weight (*Ww*) in kg and the straight fork length (*SFL*) in cm, the Fulton’s condition index (K) was calculated according to previous studies [21,22]:

(2) $$K=Ww/SFL^{3}\times 10^{5}\;$$

Muscle samples of about 1 kg per specimen were taken with a transversal cut from the anterior extremity of the upper loin, as previously described [24]. All the samples were immediately frozen in a blast chiller at −50 °C and then stored at −25 °C. A total of 30 sub-samples of raw muscle of about 500 g each were analysed in August 2020, while the remaining 30 sub-samples of about 500 g each intended for canning were stored at −20 °C.

### 2.2. Pre-Canning and Post-Canning

On February 2021, the 30 sub-samples destined for canning were thawed, cooked for 1 h (at about 100 °C) in 7% saline solution and then stored in a refrigerated cell (natural drying) for 4 days. These sub-samples were divided in half to analyse both the cooked muscles (pre-canning) and the post-canning samples canned in olive oil in half-pound boxes (equivalent to 226.8 g). After sterilization at 118 °C for 65 min, the canned samples were stored in oil for the maturation phase for no less than a year and were analysed in April 2022. The tuna meat was drained before post-canning analysis.

### 2.3. Chemical Analysis

#### 2.3.1. Determination of Total Mercury Concentration

The total mercury content was determined in compliance with US EPA 6020B. The analytical procedure involved acid digestion of the sample (1 g) in a glass vessel with 5 mL of ultrapure nitric acid, 70% (J.T. Baker, Phillipsburg, NJ, USA). The treatment was carried out in a Discover SP-D microwave digestion system (CEM Corp., Matthews, NC, USA) at a power of 600 W and a temperature fixed at 200 °C. Before instrumental analysis, the digestion solution was diluted to 50 mL and then 1 mL to 20 mL with ultrapure water MILLI-Q^®^ Quantum^®^ TEX. The instrumental analysis was performed with an inductively coupled plasma mass spectrometer ICP-MS/MS (Agilent 8800 QQQ) equipped with a collision cell and two quadrupole mass analysers. In comparison to a single-quadrupole ICP-MS system, the triple-quadrupole system greatly increases the accuracy of mass separation; 202 Hg was used as a quantification isotope and 209Bi as an internal standard element to compensate the matrix effect and signal drift. For each analysis, a method blank was carried throughout the entire sample preparation and analytical process. The calibration curve was verified at the end of each analysis using continuing calibration verification (CCV) at or near midrange. The quality control of the data was verified and controlled using Certified Reference Materials Fish muscle ERM-BB422 (mean ± s.d.: 0.601 ± 0.030 mg/kg) and Tuna BCR-463 (mean ± s.d.: 2.85 ± 0.16 mg/kg. The LOQs testing was 0.005 mg/kg for HgTOT. The method is accredited according to UNI EN ISO 17025/2017.

#### 2.3.2. Lipid Analysis

The lipid content was determined by extraction of tuna tissue with a hexane/acetone (80:20) mixture by Accelerated Solvent Extractor system ASE™ 350 (Thermo Scientific™ Dionex™, Waltham, MA, USA). A portion of 5 ± 0.1 g of homogenate tuna tissue was mixed with Hydromatrix^®^ (Agilent Technologies^TM^, Santa Clara, CA, USA). The mix was transferred to the ASE cell and extraction was performed at a temperature of 125 °C and pressure of 1500 psi. The extract was transferred to a receiver of a macro-concentration device and concentrated to near dryness. The percent lipid content was determined by weighing the residue and calculating the ratio over the initial portion of processed tuna tissue.

### 2.4. Statistical Analysis

Relationships between age class and K or muscle fat content were investigated using a one-way analysis of variance. Least squares means of the age classes were compared using a Tukey test. The correlation among body weight, muscle fat content and K was investigated using Principal Component Analysis [20]. Eigenvectors and eigenvalues were extracted and investigated in terms of the relationships with the original variables. Simple linear regressions were estimated for assessing relationships between total mercury content and body weight, muscle fat content, and K. The effect of muscle status (raw, pre-canning, canned) on mercury content was investigated using a one-way analysis of variance. Least squares means of the different muscle status were compared using a Tukey test. Statistical analysis was performed using GLM, PRINCOMP, and REG procedures of SAS software (version 9.4, Cary, NC, USA).

## 3. Results and Discussion

The biometric data of our samples, along with the results of the analysis, according to a decreasing order of weight of the specimens (and related estimate of age) are reported in Table 1.

### 3.1. Determination of Total Mercury Concentration in Raw Samples

**Figure 1:**
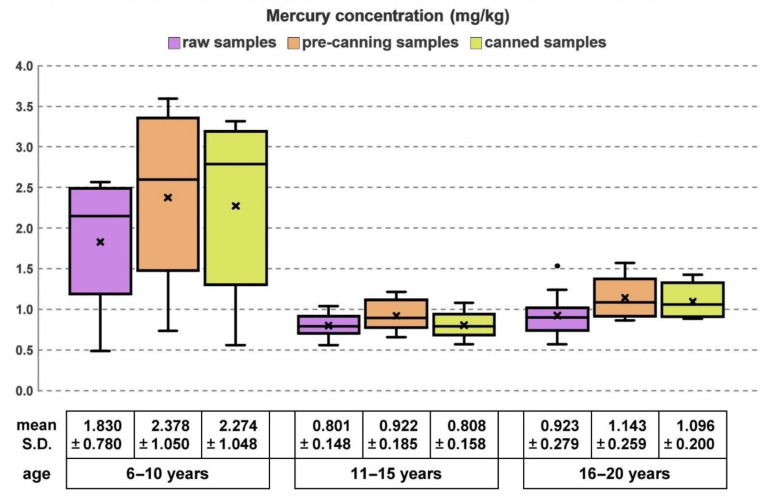
Comparison of the mercury levels in raw, pre-canning and canned samples according to age group. “X” indicates the mean values, the dot indicates an outlier.

The mean ± s.d. of mercury content in raw muscles was 1.185 ± 0.662 mg/kg (Table 1), showing a high variability of distribution, with a coefficient of variation of 55.8%. Altogether, 11 out of 30 samples exceeded the maximum tolerable mercury content in tuna. However, the highest levels of mercury were found in the younger age group (6–10 years), with a mean ± s.d. value of 1.830 ± 0.780 mg/kg (Figure 1).

On the contrary, the mean ± s.d. value of the other two age groups (11–20 years) was 0.862 ± 0.227 mg/kg. This finding appears to be in clear contradiction with the assumption that the mercury content rises with increasing weight, as this is related to age. Since several specimens of the younger class showed high levels of mercury, an apparently non-linear correlation between body weight and mercury levels for the whole age range was shown. These age classes, starting from relatively lower mercury contents, showed a positive correlation between the variable weight and the mercury contents, confirming what has already been highlighted in previous studies [33]. Another explanation should be related to the correlation between mercury levels in the muscle and the weight of the tuna, which is a function of their age, due to the phenomena of bioaccumulation over time and biomagnification along the trophic chain, since tuna are at the top of it. To explain the reasons for this unusual result, the differences between the three age groups were investigated not only in relation to the corresponding weight but also in relation to K and the percentage of fat in the muscle. The statistical analysis then produced a preliminary correlation matrix between these three principal components (Table 2).

No statistically significant differences were found between the three age groups, while a statistically significant difference regarding K (*p* = 0.0126) between the younger class and the intermediate class has been pointed out (mean ± s.d. 1.74 ± 0.08 vs., 1.91 ± 0.13). The multivariate analysis of the main components was conducted on the three variables (weight of the fish, K and percentage of fat in the muscle), and it showed (Table 3) that a first component related to all three original variables explains 59.8% of the overall variability. The second component is positively correlated to the weight of the fish and negatively to the fat content and explains 23.5%, while the latter is negatively correlated to K and explains 16.7%. 

**Figure 2:**
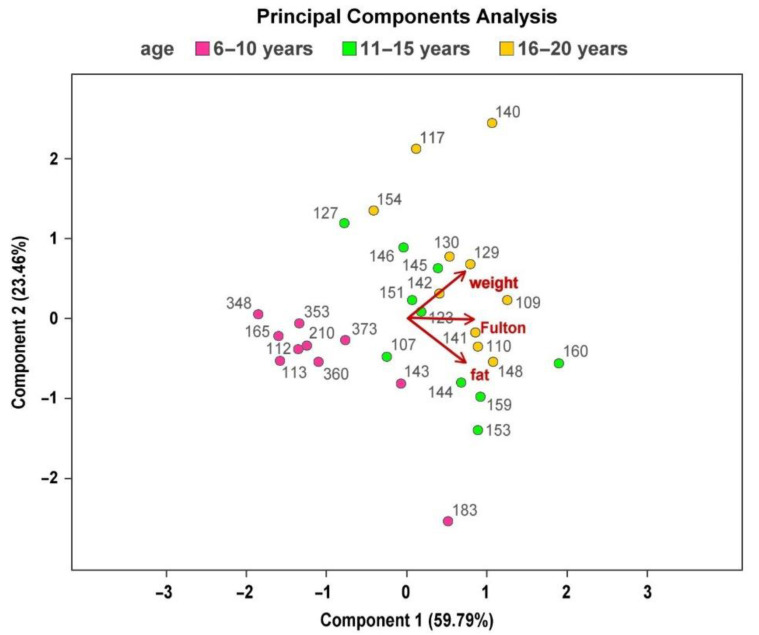
Bi-plot of the principal components analysis (PCA) carried out on the muscle fat content, Fulton’s condition index (K), and weight. Tuna of different age class have different colors.

A principal components analysis (PCA) bi-plot was also performed showing the discrimination of raw tuna samples related to muscle fat content, K and weight (Figure 2).

The individual analysis of the three variables showed that the weight of the fish in the two most advanced age groups (11–20 years) was weakly positively correlated to the mercury levels in the muscles, with a related equation (Equation (3)) in the simple linear regression model equal to

(3) $$y=0.00126x+0.53742\;\left(R^{2}=0.1204\right).$$

On the contrary, the variable K was negatively associated with the mercury levels, according to the following regression line (Equation (4)):

(4) $$y=-3.33567x+7.30673\;\left(R^{2}=0.4547\right).$$

Through the evaluation of the intercept, the critical intersection value of K with the maximum content for mercury (1.0 mg/kg of fresh weight) [13], was found to be K = 1.89. Therefore, below this value, it will be more likely to find specimens with mercury levels beyond the regulated limits. This would explain the apparently non-linear correlation observed considering only the “weight” component of the specimens and described above. The variable “percentage of fat in muscle” was also negatively associated, but to a lesser extent, with mercury levels, according to this regression line (Equation (5)):

(5) y=−0.04603x+1.85880 (R²=0.3980).

The critical intersection value at a value of 1.0 mg/kg of fresh weight was shown at 18.6% of fat in the muscle.

### 3.2. Determination of Total Mercury Concentration in Pre-Canning Samples

These results have shown that even in pre-canning Atlantic bluefin tuna, the mercury levels increase significantly compared to raw muscles. After the cooking process, the mean ± s.d. of mercury content was 1.481 ± 0.893 mg/kg (Table 1), with marked variability of distribution and with a coefficient of variation increased to 60.3%. The specific mercury concentration factor compared to raw muscle was estimated to be 0.80. According to previous studies [32], the mercury concentration could vary because a loss of moisture during cooking could cause the mercury to become more concentrated in the meat.

### 3.3. Determination of Total Mercury Concentration in Canned Samples

After the subsequent phase of canning, sterilization and maturation in oil, the mean ± s.d. of mercury content was 1.393 ± 0.882 mg/kg (Table 1), showing a further marked variability of distribution (Figure 1), with a coefficient of variation increased to 63.3%. According to previous studies on albacore tuna and skipjack tuna [5,9,10,11,12,14,25,29,30,31,32], it was also possible to estimate the specific mercury concentration factor of 0.85 compared to raw muscle (probably due to the diluting effect of the oil adsorbed by the muscle in the last phase of the process), pursuant to the aforementioned EU standard [13]. However, as previously reported [29], a large variation in the percentage increase in mercury concentration was observed. Although several studies reported constant distribution of mercury in fish tissues, the distribution of mercury in the tissues of bluefin tuna changes within myomeres [25]. The difference in the average mercury contents between the processed and the raw product was statistically significant (*p* = 0.0003). However, this difference was already statistically significant (*p* < 0.0001) among the average mercury contents of raw and pre-canning products. On the other hand, the differences between pre-canning and canned products were not significant (*p* = 0.1809). In this regard, it should be considered that the variability associated with the individual slices during the processing, from raw to cooking and then to canning and storage, lies plausibly in the residual inhomogeneity of the distribution of mercury even within each slice, as has been already shown in Southern bluefin tuna [25].

## 4. Conclusions

To the best our knowledge, this is the first study investigating the behaviour of mercury during the bluefin tuna canning process. Furthermore, in the few studies comparing fresh/frozen tuna with canned tuna, the samples were from different fish and were not followed through the canning process, and therefore it was not possible to highlight the changes in concentrations of mercury due to canning. The present study shows that, even in the case of Atlantic bluefin tuna, the canning process promotes a statistically significant increase in the concentration of mercury from raw samples to canned samples. The results of the present study showed that also in Atlantic bluefin tuna, like Southern bluefin tuna [1,2,3,4,6,7,8,9,13,15,16,17,18,19,20,21,22,23,24,25,29,31] and Pacific bluefin tuna [23], the weight of fish represents the best known, but not the only, variable associated with the dynamics of mercury accumulation in the muscles. In fact, at least two other components must be considered: K and the percentage of fat in the muscle. The correlations between these three components and the mercury content in raw, cooked, or canned samples showed high values only for the first component (weight). They are inversely associated with the accumulation of mercury in Atlantic bluefin tuna and can be indirectly estimated by competent personnel, or by means of applications that can detect in situ the length and roundness of the specimens kept alive in the trap. These components could be used as a screening tool in the selection of tunas to be directly placed on the market and/or to be fattened in aquaculture. They can help to mitigate the risk associated with the presence of mercury [1,2,3,4,6,7,8,13,15,16,17,18,19,20,21,22,23] and allow compliance with the European legislation [13].

## Figures and Tables

**Table 1:** Mercury concentration, length, weights, Fulton’s condition index (K) and lipid content in raw, pre-canning and canned samples (N = 30) of Atlantic bluefin tuna (*Thunnus thynnus*).

Age Group	Identification Number (Cod ID)	CFL * (cm)	SFL ** (cm)	Weight (kg)	Fulton’s Condition Index (K)	Lipid (%)	Mercury Concentration (mg/kg)		
							Raw Samples	Pre-Canning Samples	Canned Samples
16–20 years (*decidedly mature*)	140	270	262	372.0	2.08	3.7	0.918	0.978	1.092
	130	273	265	330.4	1.78	18.5	1.240	1.549	1.336
	117	267	259	324.0	1.87	3.1	0.894	1.117	1.068
	129	267	259	322.0	1.86	18.8	0.760	0.922	0.887
	109	262	254	318.0	1.94	23.2	0.946	1.062	1.055
	154	271	263	301.0	1.66	9.7	1.535	1.317	1.327
	148	256	248	283.4	1.86	28.5	0.570	1.157	0.915
	141	256	248	282.6	1.85	24.5	0.710	0.865	0.954
	110	252	244	271.0	1.86	25.4	0.753	0.891	0.897
	142	250	242	261.0	1.84	17.3	0.906	1.571	1.427
11–15 years (*adult*)	160	236	228	258.0	2.16	26.0	0.563	0.796	0.752
	145	245	237	253.4	1.90	12.9	0.823	0.925	0.802
	146	242	234	237.6	1.85	8.8	1.042	1.219	0.877
	153	237	229	221.4	1.83	32.2	0.728	1.115	0.939
	127	240	232	217.2	1.73	3.7	0.999	1.139	1.083
	144	229	222	206.0	1.89	24.3	0.887	0.931	0.946
	151	229	222	204.0	1.87	12.8	0.808	0.847	0.786
	123	221	214	188.0	1.92	12.9	0.741	0.873	0.615
	107	212	205	154.4	1.79	16.1	0.782	0.716	0.707
	159	192	185	134.4	2.11	19.4	0.637	0.660	0.570
6–10 years (*less young*)	143	189	183	114.8	1.89	16.1%	0.765	0.806	0.827
	360	177	171	85.0	1.70	10.5%	2.520	3.309	2.765
	373	166	160	76.2	1.86	6.6%	1.400	1.706	1.636
	183	162	156	72.6	1.91	31.0%	0.488	0.738	0.562
	165	168	162	69.8	1.64	5.7%	2.571	3.598	3.316
	112	165	159	69.4	1.72	6.9%	2.436	3.514	3.211
	348	168	162	68.4	1.61	2.6%	1.950	2.446	2.819
	113	165	159	64.8	1.61	8.6%	1.332	1.956	1.464
	353	160	154	64.4	1.75	3.3%	2.484	2.751	2.953
	210	155	149	57.4	1.72	6.3%	2.352	2.957	3.187
Mean ± s.d. ***							1.185 ± 0.662	1.481 ± 0.893	1.393 ± 0.882

Legend: * curved fork length; ** straight fork length; *** Standard deviation.

**Table 2:** Correlation matrix between muscle fat content, Fulton’s condition index (K), and weight.

	Fat	Fulton (K)	Weight
Fat	1.0000	0.4479	0.2961
Fulton (K)	0.4479	1.0000	0.4410
Weight	0.2961	0.4410	1.0000

**Table 3:** Eigenvectors and eigenvalues of the principal components (PC) of the correlation matrix of the three variables considered in the study.

	PC 1	PC 2	PC 3
Fat	0.5563	−0.6972	0.4522
Fulton (K)	0.6208	−0.0130	−0.7839
Weight	0.5524	0.7168	0.4255
Eigenvalues	59.79%	23.46%	16.74%

## Data Availability

Data is contained within the article.
